# The Relationship between Phytoplankton Distribution and Water Column Characteristics in North West European Shelf Sea Waters

**DOI:** 10.1371/journal.pone.0034098

**Published:** 2012-03-27

**Authors:** Johanna Fehling, Keith Davidson, Christopher J. S. Bolch, Tim D. Brand, Bhavani E. Narayanaswamy

**Affiliations:** 1 Scottish Association for Marine Science, Scottish Marine Institute, Oban, Argyll, Scotland, United Kingdom; 2 Systematic Biology, Department of Evolution Genomics and Systematics, Evolutionary Biology Centre, Uppsala University, Uppsala, Sweden; 3 National Centre for Marine Conservation and Resource Sustainability, Australian Maritime College, University of Tasmania, Launceston, Tasmania, Australia; Heriot-Watt University, United Kingdom

## Abstract

Phytoplankton underpin the marine food web in shelf seas, with some species having properties that are harmful to human health and coastal aquaculture. Pressures such as climate change and anthropogenic nutrient input are hypothesized to influence phytoplankton community composition and distribution. Yet the primary environmental drivers in shelf seas are poorly understood. To begin to address this in North Western European waters, the phytoplankton community composition was assessed in light of measured physical and chemical drivers during the “Ellett Line” cruise of autumn 2001 across the Scottish Continental shelf and into adjacent open Atlantic waters. Spatial variability existed in both phytoplankton and environmental conditions, with clear differences not only between on and off shelf stations but also between different on shelf locations. Temperature/salinity plots demonstrated different water masses existed in the region. In turn, principal component analysis (PCA), of the measured environmental conditions (temperature, salinity, water density and inorganic nutrient concentrations) clearly discriminated between shelf and oceanic stations on the basis of DIN∶DSi ratio that was correlated with both salinity and temperature. Discrimination between shelf stations was also related to this ratio, but also the concentration of DIN and DSi. The phytoplankton community was diatom dominated, with multidimensional scaling (MDS) demonstrating spatial variability in its composition. Redundancy analysis (RDA) was used to investigate the link between environment and the phytoplankton community. This demonstrated a significant relationship between community composition and water mass as indexed by salinity (whole community), and both salinity and DIN∶DSi (diatoms alone). Diatoms of the *Pseudo-nitzschia seriata* group occurred at densities potentially harmful to shellfish aquaculture, with the potential for toxicity being elevated by the likelihood of DSi limitation of growth at most stations and depths.

## Introduction

Although the shelf seas occupy only approximately 10°C of the world ocean, neritic phytoplankton contribute about a quarter of global primary production [1] that underpins marine food webs and regional fisheries, and represent a significant contribution to global carbon cycling [2]. Coastal waters in Northern Europe are under a range of pressures including climate driven temperature change, nutrient enrichment and pollution [3]. These and other environmental factors have been suggested to influence the temporal and/or spatial distribution of phytoplankton functional groups and even of particular species. Governing factors are potentially location specific, and it is therefore important to determine how the distribution and composition of phytoplankton populations in economically important shelf seas relate to the particular chemical and physical properties of the water column in which they live. Experiencing relatively little anthropogenic influence, the Scottish continental shelf is a potentially important reference region for policy makers implementing regulations within the EU Marine Strategy Framework and Water Framework directives. Study of this relatively un-impacted area will also provide a baseline against which to address future ecosystem change [4].

The perceived importance of harmful algal blooms (HABs) in shelf seas is increasing. The term HAB suggests that these occurrences are related to dense, perhaps mono-specific, blooms. However, lower abundance organisms, potentially occurring within multiple species communities, are also of importance. This is particularly true for those species that produce biotoxins that are accumulated by shellfish, as subsequent consumption of this shellfish may negatively affect human or animal health [5]. While most HAB species are dinoflagellates, diatoms of the genus *Pseudo-nitzschia* may also be harmful through their production of neuro-excitory toxin domoic acid (DA), which may result in amnesic shellfish poisoning (ASP) in consumers of contaminated shellfish [6]. DA may also affect marine mammals or birds that consume planktivorous fish. Other diatoms such as species of the genus *Chaetoceros* may also be harmful to farmed fish should their spines become lodged within gills [7].

Hence, as noted by Smayda and Reynolds [6] the global increase in HABs has begun to refocus interest on phylogenetic and species-level responses rather than measurements of phytoplankton functional groups or productivity. Both physical conditions and nutrient availability have been related to the growth and toxicity of biotoxin producing phytoplankton, with factors such as wind driven waters exchange [7], water temperature [8], anthropogenic nutrient supply [9], [10] and the ratio of nutrients in terms of both DIN∶DIP [11], [12] and DIN∶DSi [13]–[16] all being implicated.

The phycology of the continental shelf to the West of Scotland has received little detailed attention in comparison to other shelf-sea areas adjacent to the British Isles and elsewhere, with studies concentrating on the coastal fjordic environment [17]–[22]. Those authors that have investigated the open shelf have considered the biogeography of dinoflagellates [23]–[25], the role of localized features [26]–[28], productivity in specific locations [27], [29] or the analysis of continuous plankton recorder (CPR) data [30], [31]. While significant numbers of harmful phytoplankton events have been recorded in coastal regulatory monitoring in the region [32]–[34] these observations are typically made in isolation to other environmental variables, and hence cause and effect are not easily related.

**Figure 1:**
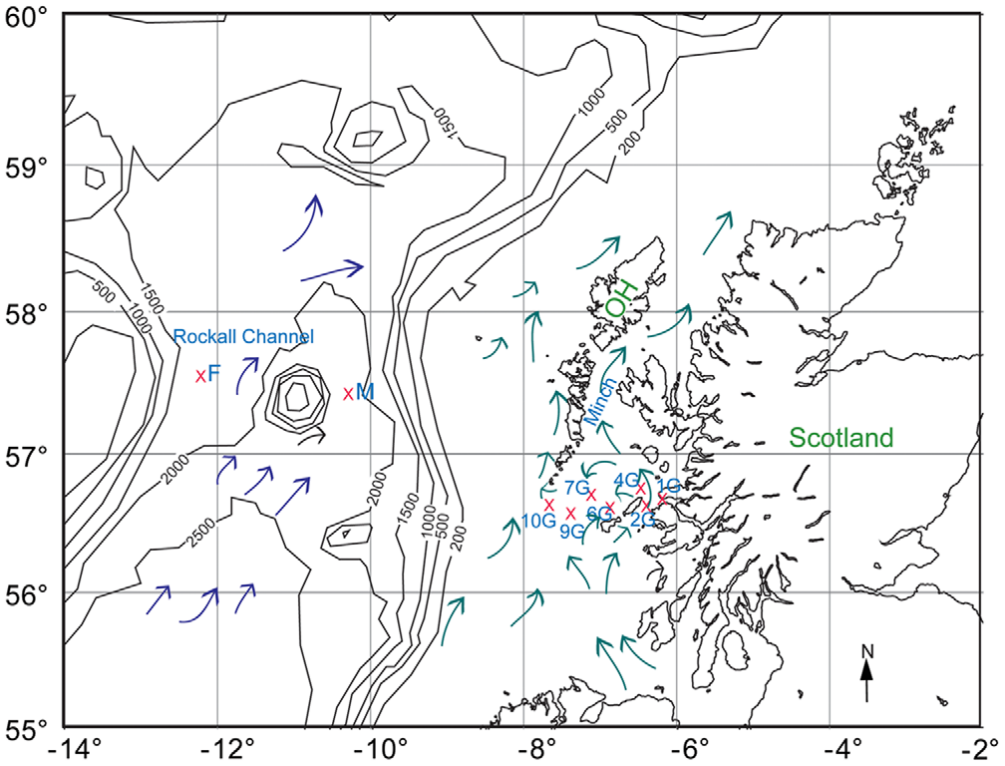
Cruise sampling stations. Ellet Line stations include shelf stations 1G–10G and open Atlantic Ocean stations M and F. Lines represent bathymetry and arrows represent circulation patterns of the Scottish coastal current and the North Atlantic surface water, redrawn after Ellett [51] and McKay et al. [50]. OH is the Outer Hebrides, the island chain lying to the west of the Minch.

Since 1975 physical oceanographic measurements have been undertaken along a transect from the Scottish west coast, starting at the Isle of Mull, leading across the Minch towards Barra Head and across the shelf to Rockall, towards the open Atlantic (Figure 1). This transect was established by David Ellett and hence has acquired the name the “Ellett Line”. Unfortunately, most studies of this transect have neglected to investigate phytoplankton populations. The only exception is Savidge and Lennon [35] who studied phytoplankton distributions during spring and summer transects in 1983, however, these data consist only of hand drawn contour plots for a limited number of “representative” species at a limited number of stations.

In this study of the Ellet Line during 2001 we therefore made the first detailed assessment of the spatial distribution of phytoplankton, and particularly the diatoms that dominated, across the north west Scottish continental shelf into the open ocean. These observations are interpreted in terms of the physical and chemical characteristics of the water column to assess the strength of environmental control of the phytoplankton population and its composition.

## Materials and Methods

### Site and Sampling details

A transect of nine stations along the Ellett Line across the Scottish Continental shelf and the adjacent region of the open Atlantic was sampled between 29 September and 4 October 2001 during RV Discovery cruise D257 (Figure 1). Samples were collected from station 1G–10G, in the inner and mid regions of the shelf. Bad weather prevented sample collection on the outer shelf or continental slope, but subsequently on the same cruise two further oceanic, deep water, stations M and F were sampled.

At each station, vertical profiles of temperature and conductivity were recorded using a Seabird 911 CTD. Water samples from up to six depths were collected with 10 L water bottles attached to the CTD. Concentrations of dissolved inorganic nitrate (DIN), silicate (DSi) and phosphate (DIP) were determined following filtration through ∼1.0 µm pore size A/E glass fiber filters (Pall Gelman), and analyzed with a LACHAT Quick chem. 8000 autoanalyser [36].

At stations, 1G, 4G, 10G, M and F (corresponding to inshore, mid shelf and oceanic water), chlorophyll *a* (chl *a*) samples were collected by filtering known volumes of water through 25 mm diameter Whatman GF/F filters and the filters were stored in the dark at −20°C prior to extraction and analysis. The samples were extracted in 5 ml of 90% buffered acetone using a soni-probe for approximately 2 minutes and the chl *a* was determined using a “simple” isocratic HPLC method for pigment followed by fluorimetric detection [37].

Two hundred and fifty ml of each water sample were preserved with Lugol's iodine (1% final concentration) for phytoplankton analysis. Phytoplankton were estimated using the Utermöhl method [38] on 50 ml subsamples and cells of greater than 5 µm diameter were counted using an inverted light microscope (Zeiss Anxiovert 100) at 200× magnification. Diatoms were identified to species level where possible. However, due to the difficulty in discriminating species within some genera by routine light microscopy, *Chaetoceros* spp. were enumerated in two size classes (cell width excluding setae > 10 µm and < 10 µm). *Pseudo-nitzschia* species were also enumerated as two size classes: 1) the *P. seriata* group (cell width > 3 µm) and 2) the *P. delicatissima* group (cell width < 3 µm) [39], [40]. Epiphytic *P. americana* were counted separately.

Due to the numerical dominance of diatoms, detailed taxonomy within our study focused on this group, however dinoflagellates were enumerated in two size classes (< 20 µm and > 20 µm) with relatively large distinctive dinoflagellates species belonging to the genera *Ceratium*, *Dinophysis* or *Prorocentrum* being identified to species.

### Statistical analysis

A marked decrease in cell density was noted below the mixed layer with most of the phytoplankton taxa not being present below 100 m depth. To ensure that only actively growing cells were included in the analysis, the mean cell density of only those cells present within the mixed layer at each station was used in the statistical analysis. Contour plots of these phytoplankton data and abiotic variables were generated using the software package Surfer (Golden Software Inc) using the minimum curvature gridding method.

Multivariate statistical techniques were applied to the data to assess the similarity of the phytoplankton community and environmental variables at the different stations and the extent to which the measured environmental variables could explain the observed cell distributions. Principal component analysis (PCA) was used to determine the similarity of measured environmental conditions between stations. Phytoplankton community abundance data were fourth root transformed to down-weight the effect of highly abundant species, and MDS plots constructed from a Bray-Curtis dissimilarity matrix to visualise similarities among the phytoplankton assemblages at each site. The PCA and MDS analyses were performed using the PRIMER™ (Plymouth Routines in Multivariate Ecological Research, Plymouth, UK). Redundancy analysis (RDA) was carried out using CANOCO™ software (Microcomputer Power, Ithaca, NY, USA) to determine the relationship between different phytoplankton, sampling stations and the measured environmental variables. Environmental and normalized (across samples) species relative abundance data were square root transformed to down-weight any extreme values.

## Results

### Hydrography

Hydrographic parameters at open ocean stations were recorded to approximately 2000 m depth. However, as phytoplankton abundance and biomass was minimal in deep water, hydrographic parameters of the open ocean stations are presented only for the top 250 m (approximately equivalent to the maximum depth of the shelf stations). The large spatial separation between the shelf and open ocean stations led us to present these data separately in some of the figures that follow.

**Figure 2:**
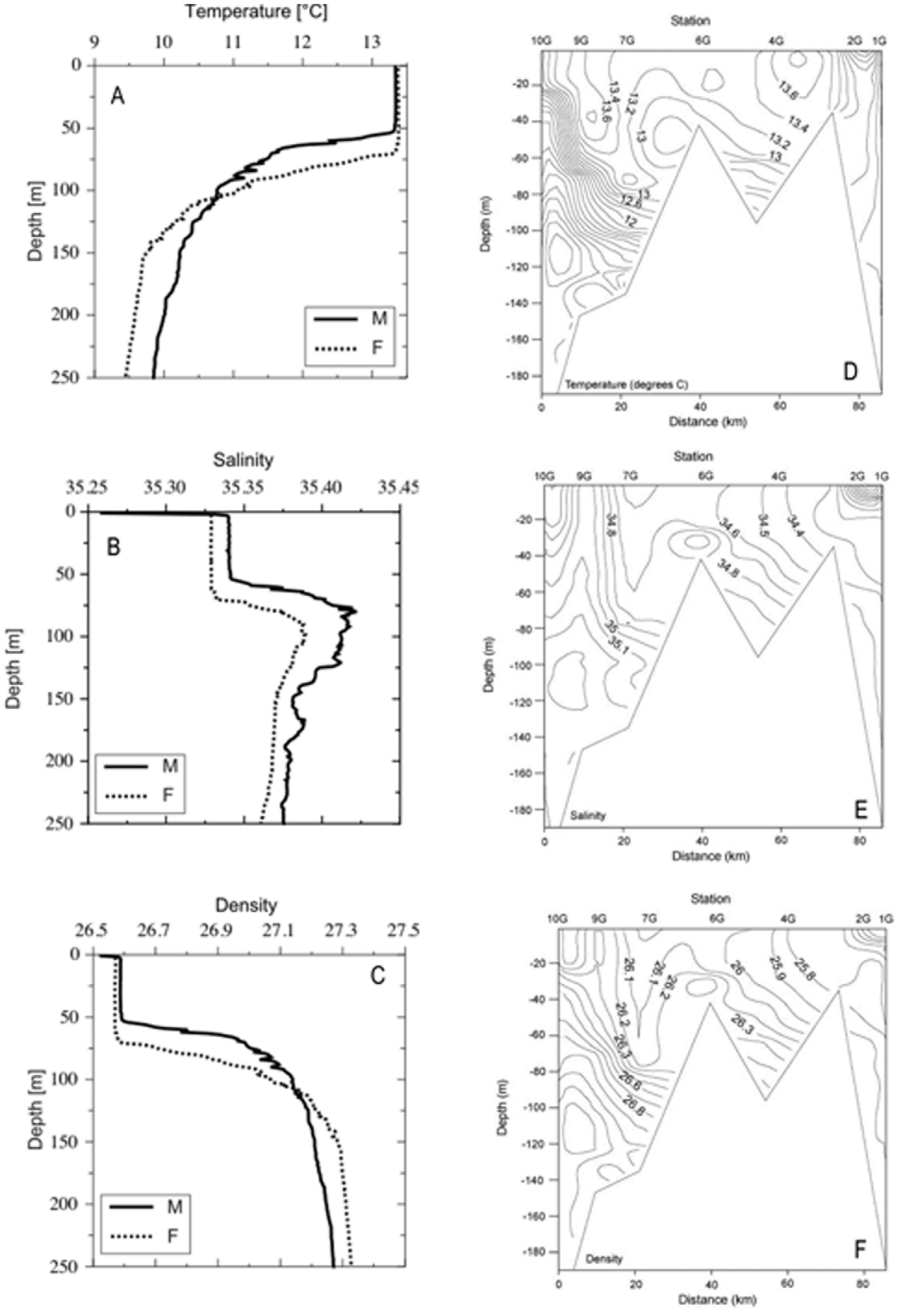
Temperature, salinity and density depth profiles and contour plots. Depth profiles of: (a) Temperature, (b) salinity and (c) density profiles within the top 250 m of at Open Atlantic stations M and F. Contour plots of (d) water temperature [°C], (e) salinity and (f) density, for shelf stations 1G to 10G.

The water temperature at open ocean stations M and F ranged from ∼13.5°C in the top 50 m to 9.5–10°C at 250 m (Figure 2). Temperature continued to decrease to a minimum of ∼3.5°C below 1500 m at both stations (not shown). A thermocline was situated at around 50 m depth. For both stations, salinity was homogeneous in the top 60/70 m with values in surface waters around 35.33 (M) and 35.34 (F), increasing rapidly to values of 35.41 (M) and 35.39 (F) in the halocline below. The temperature and salinity derived density profile (σ_t_), Figure 2c, ranged from 26.53 and 26.57 at stations M and F respectively in the top 30 meters to 27.26 (M) and 27.33 (F) at 250 m, with the pycnocline situated about the same depth as the thermocline.

At inshore stations 1G to 6G the water temperature (Figure 2d) was relatively homogenous with station and depth at ∼13°C, although a small thermocline existed at station 4G from about 75 m. A more pronounced thermocline was also evident at all stations to the west of station 6G, the depth of which decreased as one moved offshore.

Salinity of shelf waters (Figure 2e) increased as one moved offshore. At 1G and 2G slightly fresher water (presumably runoff from land), with salinities between 34.2 and 33.8, lay on top of the otherwise well mixed water column. 4G and 6G were well mixed. At 7G water with higher salinity (above 35), was found at about 90 m. The water column at 9G was well mixed down to about 80 m and showed a salinity again higher than 35. At 10G salinity increased slowly with depth and no strong gradients were observed.

Salinity readings were reflected in the water density, with σ_t_ of the surface water at 1G being reduced (Figure 2f). Water below 50 m at 4G showed a density above 26.3, indicating it belonged to the same water mass that was found at 10G, 9G and 7G at approximately 30 m, 40 m and 70 m, respectively. Below those depths, stations 10G, 9G and 7G were clearly influenced by high salinity water.

**Figure 3:**
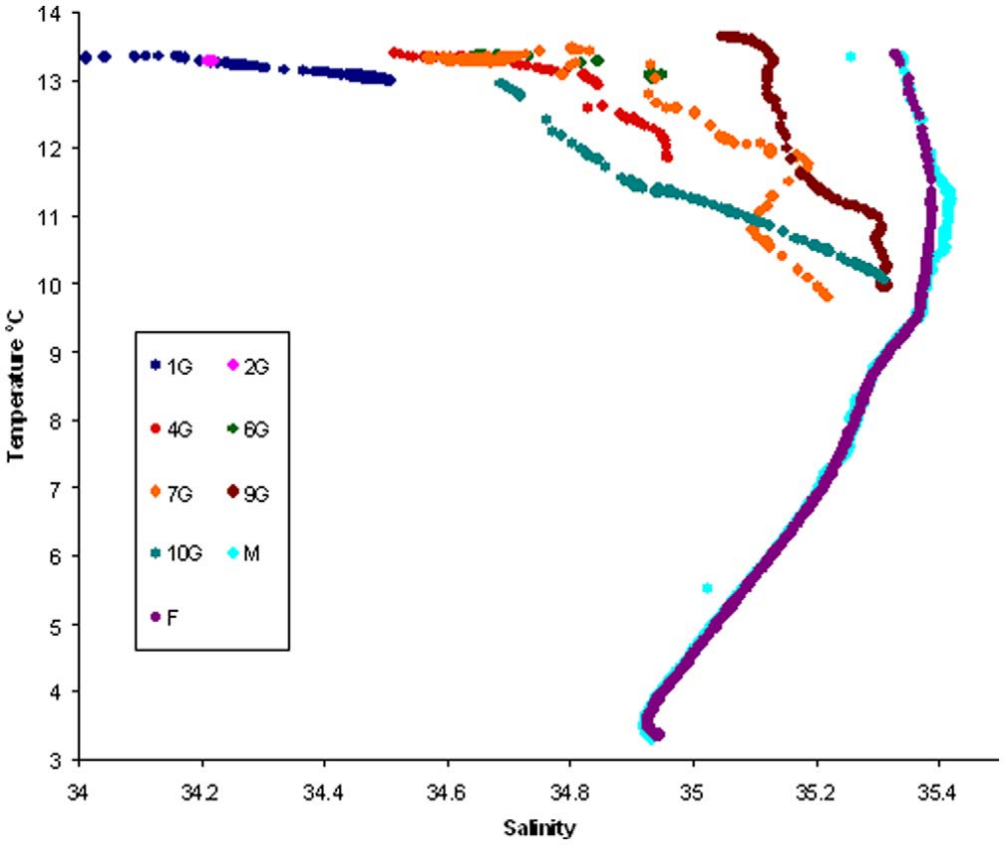
Temperature-salinity diagram of all sampled stations.

The different water masses encountered in the study can be best visualized using a temperature/salinity (T/S) diagram (Figure 3). As indicated by their overlapping T/S contours, oceanic stations M and F lie within the same water mass which is clearly distinct from the water of the shelf stations. However, the proximity of the T/S contours of 10G, 9G and 7G to M and F indicated that the bottom water of these offshore shelf stations shared the same origin as the oceanic stations. Considerable differences were evident between stations 9G and 10G at shallower depths with the upper water column at 10G resembling the mixed water of stations 1G in 170 m and 4G from about 45 m depth. In contrast, the surface waters of 9G were much more similar in character to the oceanic water of M and F.

In mid shelf waters (4G, 6G and 7G), T/S contours indicated a general similarity between water masses. Finally, the T/S characteristics of the most coastal stations (1G and 2G) coincided, indicating their location within the same water mass. Station 2G was represented by a single point in the T/S diagram indicating a well mixed homogeneous water column at this station, the relatively shallow water depth (26 m), allowing the full depth wind or tidal mixing.

### Inorganic nutrients

**Figure 4:**
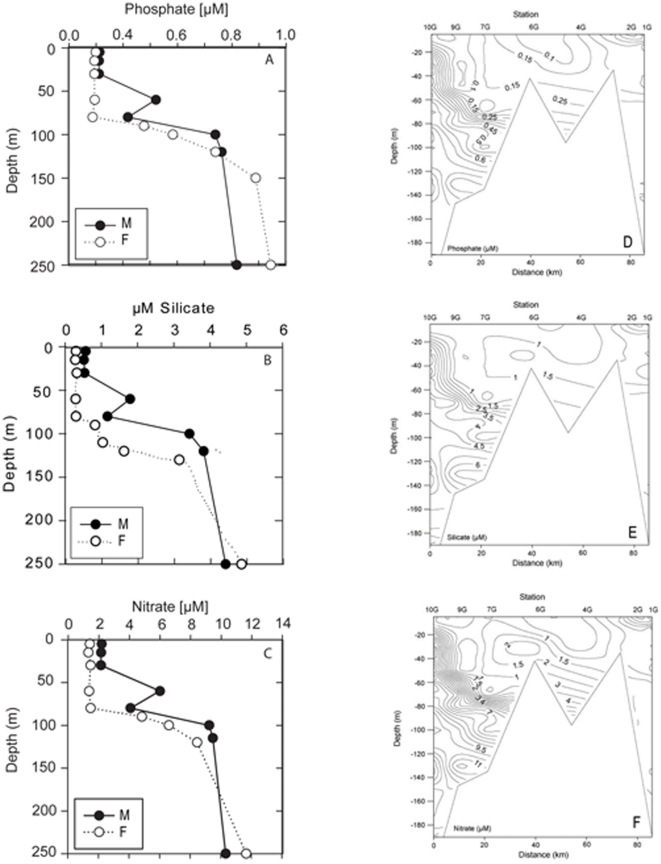
Depth profiles and contour plots of nutrients at stations M and F. Depth profiles of: (a) dissolved inorganic phosphate (DIN), (b) silicate (DSi) and (c) nitrate (DIN) concentrations in µM within the top 250 m at stations M and F. Contour plots of (d) dissolved inorganic phosphate, (e) dissolved inorganic silicate, (f) dissolved inorganic nitrate, for shelf stations 1G to 10G.

Inorganic nutrient concentrations at open ocean stations M and F generally increased with depth (Figure 4). DIN, DSi and DIP concentrations were low in water shallower than ∼50 m (< 2.5 µM DIN, <0.5 µM DSi, and <0.1 µM DIP respectively). Thereafter all increased until about 100 m (∼9 µM DIN, 1–4 µM DSi, 0.6 µM DIP). Subsequently, concentrations continued to increase more slowly with depth to maximum values of ∼16 µM DIN, ∼16 µM DSi, and ∼1 µM DIP in deep water (not shown).

On the shelf, nutrient concentrations in surface waters were also low (Figure 4), with typical values of 0.25–1.5 µM DIN and 0.63–1.5 µM DSi, and 0.1–0.2 µM DIP. Nutrient concentrations at the inshore stations 1G to 6G, were either homogeneous (station 2G) or increased only slowly with depth. However, the increase in DIN concentration in water deeper than 50 m at station 1G was markedly less than other stations. Further offshore (stations 7G–10G), a nutricline was evident that broadly corresponded to the thermocline (compare Figure 2c). Below this, the maximum nutrient concentrations were 11 µM DIN, 6.5 µM DSi, 0.8 µM DIP.

**Figure 5:**
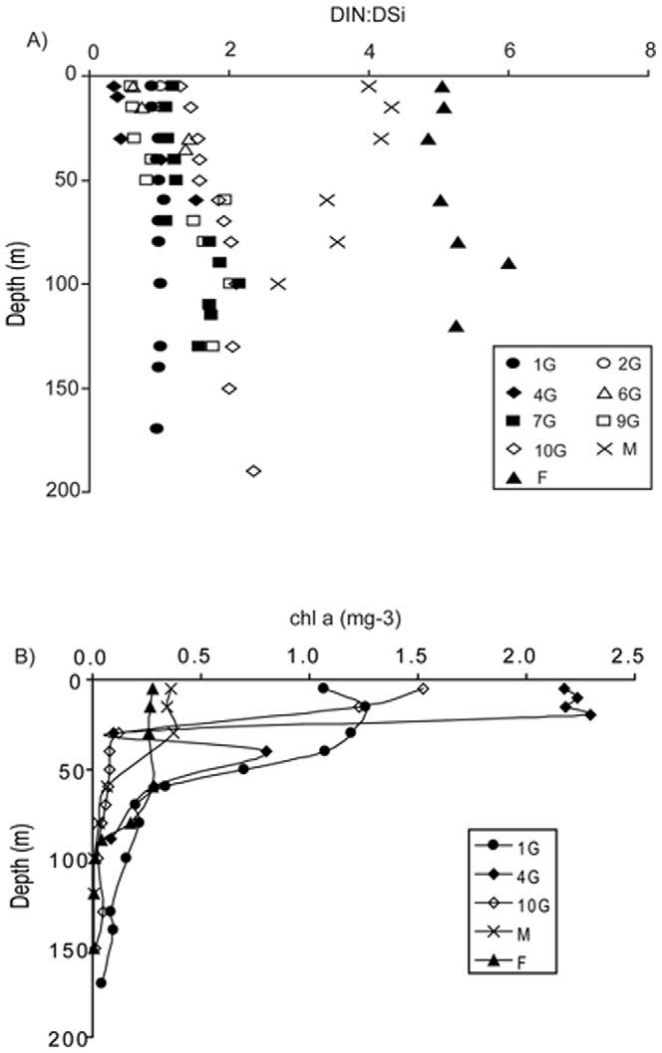
N∶Si ratio and Chl *a* profile. (a) N∶Si in the water column as a function of depth at all stations, (b) Chl *a* depth profile in mg m^−3^ at the two open ocean and three of the shelf stations.

Consistent with the low DSi concentrations at stations M and F, the DIN∶DSi ratio at these stations markedly exceeded 1 at all depths less than 200 m (Figure 5a). However, at coastal stations this ratio exhibited a more complex pattern, exceeding 1 at stations 2G, 7G and 10G. At stations 1G and 9G the ratio was slightly below 1, but at stations 4G and 6G it was markedly less than 1 in shallow water, but increased rapidly at intermediate depth. Except at shallow depths at stations 4G, 6G and 9G, the DIN∶DSi ratio exceeds the 1∶1 ratio suggested to represent the average utilization of diatoms [41]. The DIN∶DSi ratio correlated positively with salinity (r  =  0.44, p  =  0.004), more strongly with density (r  =  0.65, p  =  0.000) and (negatively) with temperature (r  =  0.78, p  =  0.000).

### Chlorophyll a

In all cases, the highest concentrations of chl *a* were in surface waters, with chl *a* concentrations falling to zero at or below 100 m for all but the most coastal station, 1G. (Figure 5b). Highest chl *a* concentration (2.3 mg chl *a* m^−3^) was found at 20 m at station 4G. Chl *a* concentrations in oceanic water were considerably lower than those on the shelf, with a maximum of 0.4 mg chl *a* m^−3^ at 30 m at station M.

### Phytoplankton

The most abundant phytoplankton genus encountered was the diatom *Chaetoceros*, comprising 69% of all cells enumerated (Table 1). Other organisms that comprised more that 1% of total abundance were the diatoms groups *Pseudo-nitzschia delicatissima* and *Pseudo-nitzschia seriata*, dinoflagellates both greater and less than 20 µm, and the diatom species *Lauderia annulata*, *Asterionellopsis glacialis*, *Eucampia zodiacus*, *Thalassiosira* sp., and *Leptocylindrus danicus*.

**Table 1:** Enumerated phytoplankton species.

Organism	On shelf mixed layer mean abundance [cells L^−1^]	Off shelf mixed layer mean abundance [cells L^−1^]	Maximum mixed layer mean abundance [cells L^−1^]	Location of maximum mixed layer mean abundance
**Diatoms**				
*Chaetoceros* spp. < 10 µm	10,4621	50	256,242	4G
*Chaetoceros* spp. > 10 µm	39,785	53	192,473	2G
*Lauderia annulata*	24,875	0	56,410	4G
*P.seriata* group	8,466	18	18,780	2G
*Asterionellopsis glacialis*	3,386	0	8,440	1G
*Eucampia zodiacus*	3,086	0	5,853	2G
*Thalassiosira* spp.	2,768	365	6,393	4G
*P. delicatissima* group	1,602	4,285	4,340	F
*Leptocylindrus danicus*	2,549	0	4,603	4G
*P. americana*	1,467	0	2,625	10G
*Guinardia delicatula*	937	8	1,404	1G
*Rhizosolenia setigera*	574	118	1,580	10G
*Cylindrotheca closterium*	490	455	1,160	2G
*Thalassionema nitzschioides*	447	0	1,387	2G
*Ditylum brightwellii*	241	0	475	1G
*Rhizosolenia styliformis*	189	85	920	4G
*Dactyliosolen* sp.	1	375	545	M
*Paralia sulcata*	102	0	273	2G
*Pleurosigma* sp.	53	23	93	2G
*Guinardia striata*	47	0	213	6G
*Corethron* sp.	44	0	127	2G
*Skeletonema costatum*	18	0	57	4G
*Meuniera membranacea*	14	0	48	7G
*Stephanopyxis turris*	7	0	24	4G
**Dinoflagellates**				
Dinoflagellates < 20 µm	2,504	9,413	11,210	F
Dinoflagellates > 20 µm	6,216	1,213	3,257	4G
*Ceratium lineatum*	682	30	1,520	2G
*Prorocentrum micans*	156	0	370	1G
*Ceratium fusus*	61	80	167	2G
*Ceratium furca*	67	33	247	2G
*Dinophysis acuta*	51	0	107	2G
*Ceratium tripos*	20	28	55	F
*Ceratium horridum*	23	2.5	60	6G
*Dinophysis acuminata*	19	0	47	2G
*Dinophysis norvegica*	12	5	53	4G
*Ebria tripartita*	10	5	33	2G
**Silico flagellates**				
*Dictyocha speculum*	61	15	235	1G

The station averaged abundances are the mean of mean mixed layer abundances at different stations. The maximum mean abundance was calculated by summing cell density in the mixed layer a particular station and dividing by the number of depths sampled. The full taxonomic data set is available in File S1.

**Figure 6:**
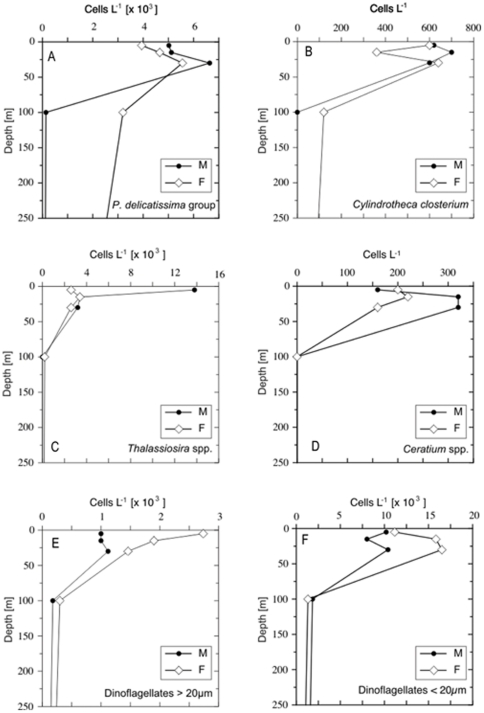
Depth profiles of cell densities off shelf. Abundances of the most abundant diatoms and dinoflagellates at station M and F within the top 250 m.

**Figure 7:**
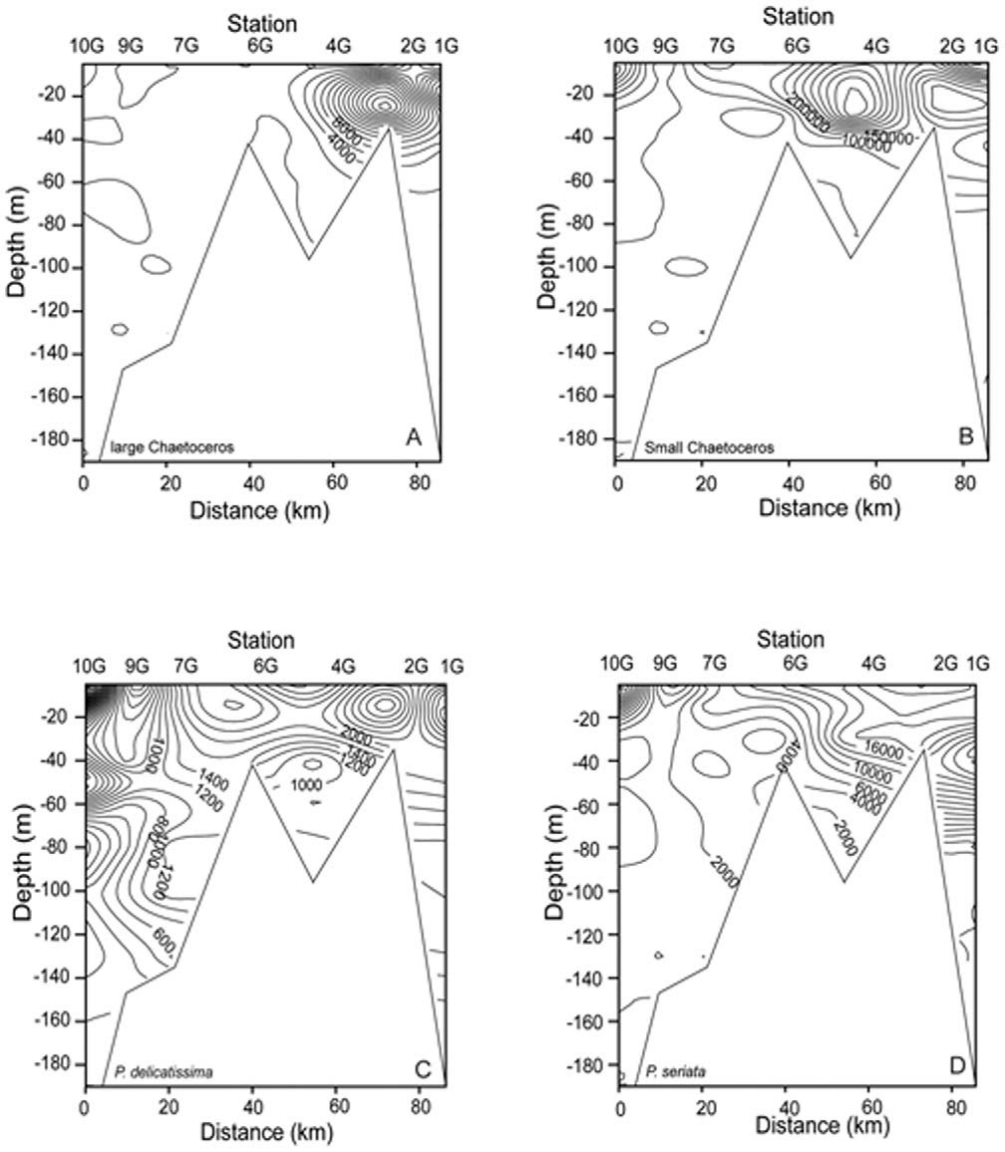
Contour plots of *Chaetoceros* and *Pseudo-nitzschia* on shelf. Vertical and horizontal distribution [cells L^−1^] of (a) large *Chaetoceros* spp. (b) small *Chaetoceros* spp. (c) *P. delicatissima* group (d) *P. seriata* group abundance, at the shelf stations (10G to 1G).

**Figure 8:**
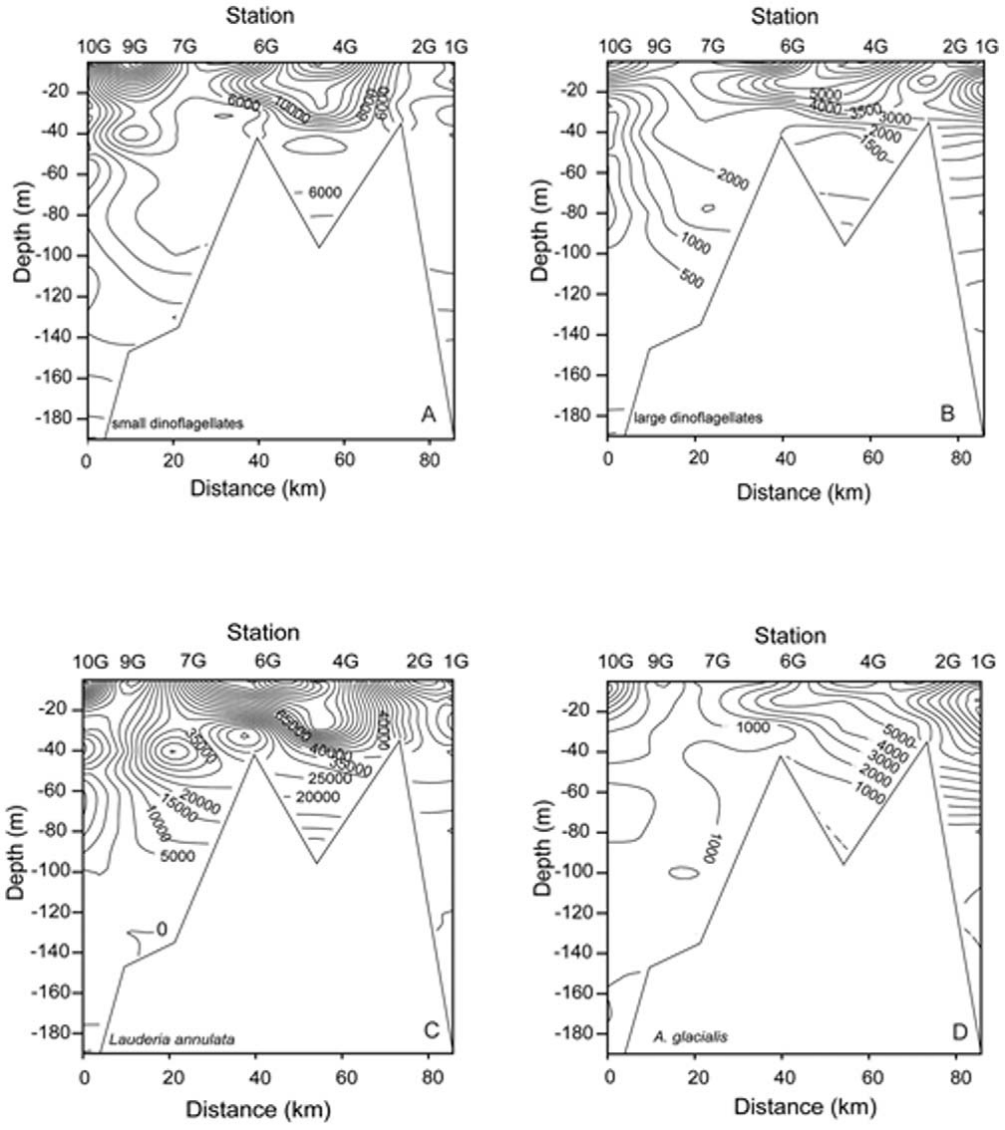
Contour plot of *Lauderia annulata* and *Asterionellopsis glacialis* on shelf. Vertical and horizontal distribution [cells L^−1^] of (a) dinoflagellates < 20 µm. (b) dinoflagellates > 20 µm, (c) *Lauderia annulata* and (d) *Asterionellopsis glacialis*, at the shelf stations (10G to 1G).

The distribution of phytoplankton across sites was far from homogeneous (Table 1, Figures 6, 7, 8). For example *Chaetoceros* spp. < 10 µm were most prevalent at station 4G (with a depth averaged mean abundance of ∼2.6×10^5^ cells L^−1^ at that station), while *Chaetoceros* spp. >10 µm were most abundant at 2G (∼71% of cells at this station). Most of the enumerated taxa were observed in highest mean station abundance at the coastal stations 1G (4 groups) and 2G (15 groups). Nine groups exhibited maxima at station 4G somewhat further offshore (Table 1). No groups exhibited their maximum abundance at station 9G.

### Phytoplankton at Oceanic stations M and F

There were marked differences in the phytoplankton composition between the oceanic and shelf stations. Only diatoms of the *P. delicatissima* group, *Dactyliosolen* sp., the *P. seriata* group (in very low numbers), *Chaetoceros* spp., *Cylindrotheca closterium*, *Guinardia striata* (only three cells), the two *Rhizosolenia* species, *Thalassiosira* spp., the two dinoflagellates size classes, all *Ceratium* spp, *Dinophysis norvegica*, *Dictyocha speculum* and *Ebria tripartita* occurred at the open ocean stations. Of these organisms, only four groups (dinoflagellates >20 µm, *P. delicatissima* group, *Dactyliosolen* sp. and *Ceratium tripos*) had their maximum abundance off the shelf. *Dactyliosolen* sp. was the only organism present solely off the shelf.

In general, cell densities of the most abundant groups were lower than at the shelf stations, with the exception of the *P. delicatissima* group (Figures 6, 7). The depth profiles were generally similar between the two oceanic sites for both diatoms and dinoflagellates with a peak in abundance at the surface or at shallow depth of 10–30 m (Figure 6). However, *Thalassiosira sp.* were considerably more abundant in the surface waters of station M and both <20 µm, and >20 µm dinoflagellate groups were more abundant at station F than M. At both stations small gymnodinoid -like species were common and *Ceratium* species (not shown) were found in maximal numbers in 15 m depth and no cells were found below 30 m. Although their cell numbers were relatively low (maximum of 320 cells L^−1^, 15 m at M), cells of these species were amongst the largest encountered in this study, therefore their biomass may be a significant fraction of the total.

### Phytoplankton distribution on the shelf

Highest densities of the abundant *Chaetoceros* spp. were usually found between 5 and 30 m, with maxima at stations 2G (25 m, 3.12×10^5^ cells L^−1^) and 4G (30 m, 5.61×10^5^ cells L^−1^) for large and small cells respectively (Figures 7a,b). Large *Chaetoceros* were particularly associated with the three most coastal stations. Peak <10 µm *Chaetoceros* densities at station 4G correspond to the highest measurements of chl *a*. The smaller size class exhibited a similar, although less pronounced pattern, with a further increase at station 10G.

The potentially toxic *P. seriata* group were more homogeneously distributed, exhibiting maximum concentration (more than 2.6×10^4^ cells L^−1^) at station 1G and 40 m depth. Concentration decreased throughout the water column and offshore to 9G, but with an increase at 10G (Figure 7d). The distribution of the *P. delicatissima* group (Figure 7c) differed from that of the *P. seriata* group, with maximum density on the shelf close to the coast (station 2G) in shallower water (∼25 m) and marked increase at the furthest offshore shelf station, 10G. The spatial distribution of the other most abundant diatoms *Lauderia annulata* and *Asterionellopsis glacialis* are presented in Figure 8c,d, exhibiting concentration maxima at station 4G and lowest concentrations at 9G.

Dinoflagellates of both small and large size classes were most abundant at stations 4G and 6G in the top 40 m of the water column (Figure 8 a, b). In marked contrast to most diatom taxa, there was also a density maximum at station 9G (particularly the <20 µm size class). The distribution of *Ceratium* spp. (not shown) was generally similar to that of the (large and small) *Chaetoceros* species, with highest density in 15 m at 4G and decreasing abundance with depth and offshore towards 9G.

### Statistical analysis

**Figure 9:**
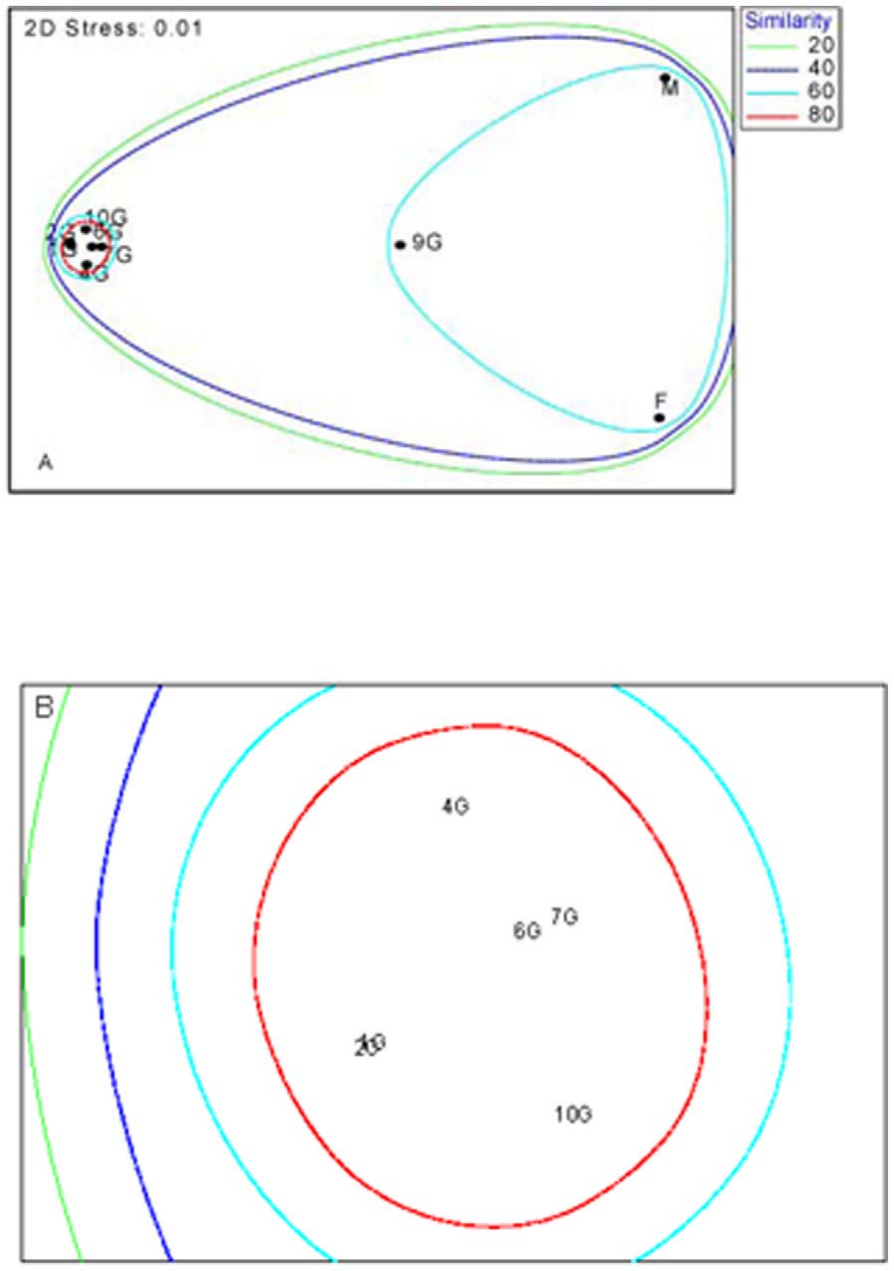
MDS ordinations of environmental variables. MDS ordination of stations along the Ellett Line transect, sampled during D257. Samples above 100 m depth were included, data were fourth root transformed. (a) all stations, (b) expanded view of stations 1G, 2G, 4G, 6G, 7G, 10G.

The MDS analysis of phytoplankton species similarity between stations exhibited a low stress factor of 0.01 indicating that the ordination is a good two dimensional representation of the multidimensional species space (Figure 9a). The ordination clearly separated the assemblages at the offshore stations M and F. While the majority of shelf stations (1G, 2G, 4G, 6G, 7G, 10G), which span a distance of only 90 km, clustered together with at least 80% similarity. An expanded view (Figure 9b) demonstrates that their ordination broadly reflected their geographical location, with stations 1G/2G and 6G/7G being very closely related and stations 4G and 10G being distinct from these two groupings (Figure 9b). Station 9G was an exception to this pattern being more similar to stations M and F (Figure 9a).

**Figure 10:**
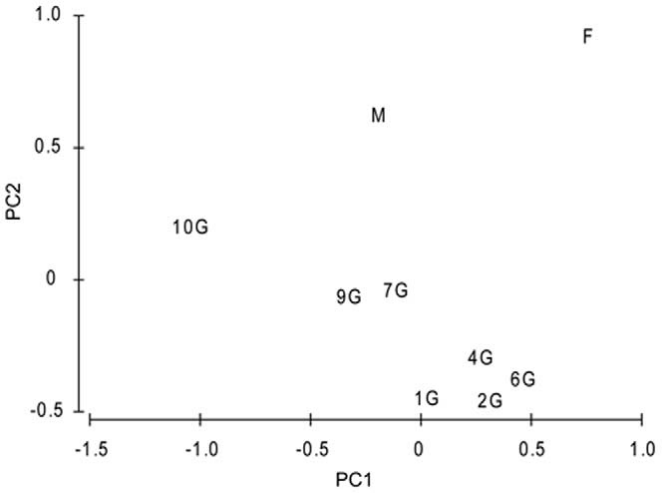
PCA ordination of environmental variables. Principal component analysis normalized environmental data from stations along the Ellett Line transect. Parameters included are temperature, salinity, density, phosphate, silicate and nitrate from the upper 100 m of the water column. The majority (60.4%) of the variance in the data was explained by the principal component 1 (PC1), while component 2 (PC2) accounted for 36.9%, giving a total explained of variation 97.3% from these two axes alone (Table 2).

The PCA ordination of the sampled stations according to the measured environmental parameters is plotted in Figure 10 with eigenvalues presented in Table 2. The analysis indicated general geographical groupings of stations in terms of their physical/chemical characteristics with near shore stations 1G, 2G, 4G and 6G being the most similar. Stations 7G and 9G were somewhat separate from this first grouping and station 10G was the most distinct of all shelf stations. Within PC1, the DIN, followed by DSi had the greatest influence on the ordination (Table 2). PC2 discriminated the shelf stations from the open ocean stations and was most strongly related to the ratio of DIN and DSi (that was in turn correlated with salinity and temperature). The diagonal trajectory of shelf stations within the ordination indicated that a combination of PC1 and PC2 best separated these.

**Table 2:** PCA eigenvalues.

Variable	PC1	PC2	PC3	PC4	PC5
DIN	−0.747	0.417	0.480	0.164	0.058
DSi	−0.599	−0.257	−0.603	−0.199	−0.409
DIP	−0.171	−0.013	−0.369	0.493	0.465
Temperature	0.127	−0.070	0.172	0.614	−0.725
Salinity	−0.009	0.063	0.189	−0.376	−0.215
Density	−0.025	0.066	0.147	−0.415	−0.097
DIN∶DSi	0.193	0.864	−0.428	−0.022	−0.178

Eigenvalues of the environmental variables: coefficients in the linear combinations of variables making up principal components of PCA.

**Figure 11:**
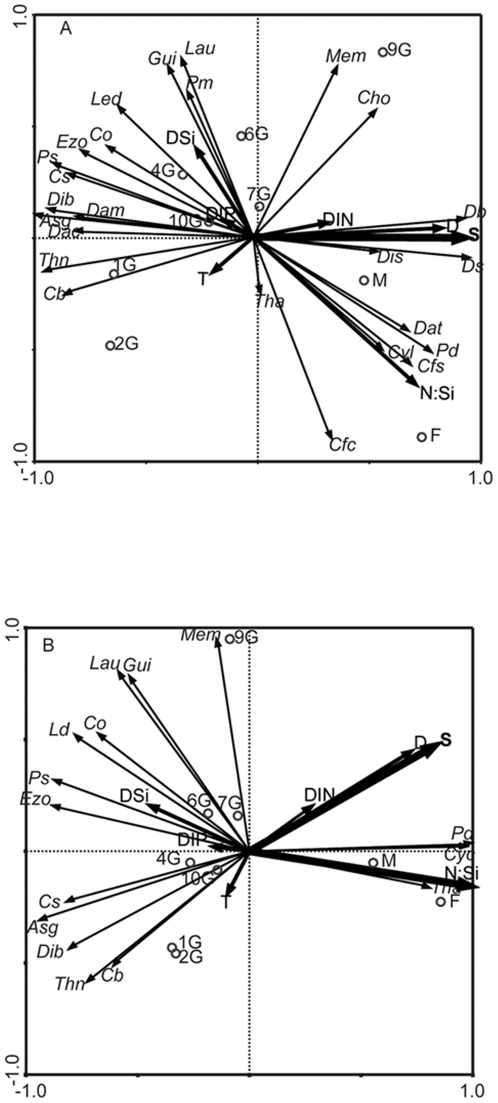
RDA correlation triplots of the relationship between phytoplankton and environmental variables. RDA of samples taken in the upper 100 m of the water column along the Ellett Line transect. a) whole phytoplankton assemblage data b) diatom species only. Analysis was based on the relative phytoplankton abundance (standardized by norm). Sampled stations are indicated by circles, phytoplankton species and environmental variables are represented by arrows with bold typeface being used for those environmental variables that were statistically significant. Environmental abbreviations: S: salinity, T: temperature, D: density, DIN, DIP, DSi: concentrations of inorganic nitrogen, phosphorous and silicon respectively, N∶Si: the inorganic DIN∶DSi ratio. Species abbreviations: *Asg*, *Asterionellopsis glacialis*, *Cb*, *Chaetoceros* spp. > 10 µm; *Cfc*, *Ceratium furca*; *Cfs*, *Ceratium fusus*; *Cho*, *Ceratium horridum*; *Co*, *Corethron* sp.; Cs, *Chaetoceros* spp. < 10 µm; *Cyl*, *Cylindrotheca closterium*; *Dac*, *Dinophysis acuta*; *Daf*, *Dactyliosolen* sp.; *Dam*, *Dinophysis acuminata*; *Db*, Dinoflagellates > 20 µm; *Dib*, *Ditylum brightwellii*; *Dis*, *Dictyocha speculum*, *Ds*, Dinoflagellates < 20 µm; *Ezo*, *Eucampia zodiacus*; *Gui*, *Guinardia striata*; *Lau*, *Lauderia borealis*; *Led*, *Leptocylindrus danicus*; *Mem*, *Meuniera membranacea*; *Pd, P. delicatissima* group; *Prm*, *Prorocentrum micans*; *Ps*, *P. seriata* group; *Tha*, *Thalassiosira* sp.; *Thn*, *Thalassionema nitzschoides*.

RDA analysis of the complete species assemblage data indicated that, of all measured environmental parameters, only salinity (Monte Carlo test, p  =  0.002) had a statistically significant impact on the composition and distribution of the phytoplankton assemblage (Figure 11a). The sum of all canonical eigenvalues indicated that 89% of the observed species variation was accounted for by the environmental variables. Open ocean, high salinity, stations M and F were placed to the right in the ordination, correlated with salinity. Consistent with the T/S and MDS plots, station 9G was only weakly related to the other shelf stations and placed intermediate between these and the open ocean stations in the ordination. Stations 10G to 4G were situated between x-axis positions −0.5 to 0, consistent with intermediate salinity conditions, while coastal stations 1G and 2G were negatively correlated with salinity. The analysis demonstrated a clear demarcation between groups of species. Large and small dinoflagellates correlated closely with salinity, whereas those diatoms that were abundant at off shelf sites were closely related to the N∶Si ratio. RDA analysis of the complete assemblage at the shelf stations alone (not shown) also found salinity to be the most important variable but with a reduced level of significance (p =  0.07).

A further RDA analysis with diatom only data (Figure 11b), showed that salinity remained significant (p =  0.004) but that N∶Si ratio was now also a significant predictor (p =  0.012). In this case, the sum of all canonical eigenvalues indicated that 91% of the observed variation was accounted for by the explanatory variables. RDA analysis of diatoms at the shelf stations alone indicated that salinity (p =  0.001) and DIN (p =  0.048) were related to the diatom distribution (not shown).

## Discussion

### Phytoplankton biomass and functional groups

Phytoplankton biomass concentrations, as assessed by chl *a*, were typical of the region in autumn [32], with our peak chlorophyll concentration of 2.3 mg chl *a* m^−3^ being approximately half the value reported by Savidge & Lennon [35] during their August study of the same transect. While the Scottish North West coast is relatively un-enriched in terms of nutrients, with total N input being less than 3 kt/year from 1990 to 2006 and much lower than the more enriched catchments of the Clyde or the east coast that regularly exceed 15 kt/year [42], some terrestrial impact is still likely with the potential to accelerate phytoplankton growth and elevate phytoplankton biomass, particularly in near coastal waters [43]. However, the greatest phytoplankton biomass was found somewhat offshore, at station 4G. Most probably this was related to the relatively greater salinity and density of this station compared to the more coastal stations of 1G and 2G. Chl *a* also exhibited its maximum in near surface waters in all locations rather than, for example, at a deep chlorophyll maximum. This suggests that the reduced irradiance of autumn was a key factor in determining the shallow depth distribution of the observed phytoplankton.

Within the phytoplankton biomass there were marked differences in the distribution of the functional groups (diatoms/dinoflagellates) enumerated. Consistent with the observations of Savidge & Lennon [35] in spring/summer we found diatoms to dominate on the shelf, and dinoflagellates to exhibit their maximum abundance off the shelf. However, while the abundance of off shelf dinoflagellates was markedly greater, this abundance based pattern was less clear at the level of group or species, as >20 µm dinoflagellates, and the larger individual dinoflagellate species enumerated, exhibited maximum abundance at shelf stations. High dinoflagellate concentrations are often associated with vertical stability and hence intermediate to strong stratification of the water column [23], [44], yet the low abundance of dinoflagellates at most shelf stations cannot be easily related to the relatively well mixed conditions of the autumn, as mixing was also strong at the offshore stations where these organisms dominated. However, Smayda & Reynolds [6] proposed that dinoflagellates are ecologically diverse with different groups of organisms adapted to different levels of nutrient, mixing and euphotic zone depth. Our observations of small gymnodinoid -like species off-shelf in autumn, at a time of decreased irradiance and increased mixing depth, support their dinoflagellate distribution turbulence/nutrient matrix as does the presence of larger organisms such as *Dinophysis* spp. and *Ceratium* spp. in the shelf-based coastal current.

### Diatom species composition

The low abundance of diatoms at oceanic stations is consistent with the low DSi concentrations (<0.5 µM) that were evident until well below 50 m at these stations. DSi concentrations were also relatively low at on-shelf stations (typically < 1 µM in the top 50 m), and less than the frequently quoted 2 µM threshold suggested by Egge & Asknes [45] to be necessary for diatoms to dominate. Time lags may occur between nutrient utilization and phytoplankton growth [46], and therefore while the DSi concentration may have been depleted by the diatom bloom, our study is consistent with others in the region, that have observed substantial diatom populations at low DSi concentrations over an extended temporal period [19].

Smayda & Reynolds [6] suggest that diatoms are cosmopolitan, exhibiting species rich pools and limited habitat speciation. While the 80% similarity of all but one of the shelf based stations in our MDS analysis leads us to concur with this argument in general terms, we still found considerable spatial diversity within the diatom assemblage as outlined below.

The numerical dominance of the phytoplankton community by species of *Chaetoceros*, is consistent with the observations of Savidge & Lennon [35] from the spring and summer of 1983. Savidge & Lennon [35] do not discriminate between size classes or species, but we found the *Chaetoceros* community to be quite mixed with no single dominant species in either size class.

Within the *Pseudo-nitzschia seriata* group only *P.* cf. *subpacifica* was present in open ocean samples. While diatoms belonging to the *P. seriata* group have been observed previously (June 1996) in oceanic waters in the region (at 59°N, 20°W) [47], they were enumerated only as the as the ‘*Nitzschia seriata* complex’ and hence no comparison of species can be made. On shelf, the *Pseudo-nitzschia* seriata group was more abundant. Of the two main *Pseudo-nitzschia* groups, only this one has been confirmed to contain toxin producing species in Scottish waters (*P. australis* [48] and *P. seriata* [40]). Our results are therefore in contrast with the limited early observations (in 1983) that found the *P. seriata* group to be rare or absent from the shelf [35]. Given that spatially and temporally elevated DA toxin levels generated by P*seudo-nitischia* have frequently been found in offshore harvested scallops in this region [40], [48] it seems likely that the *Pseudo-nitzschia seriata* group is a frequent member of the shelf flora.

An exception to the typical on/off shelf distribution of diatoms in the study was the *Pseudo-nitzschia delicatissima* group. Although, in general, density of the *P. delicatissima* group was relatively low (∼34% of *P. seriata* group density), it exhibited higher density at stations M and F than on the shelf. Savidge & Lennon [35] found the *Nitzschia* group to dominate their off shelf stations in spring but to be replaced by *Chaetoceros* in summer. Our observations suggest that further species succession occurs in autumn, returning to the spring pattern of dominance. A time series study of *Pseudo-nitzschia* in near coastal waters [19] demonstrated a regular bloom of the species *P. delicatissima* in spring and a more mixed assemblage from within the *P. delicatissima* group in summer. However, consistent with our results here, an autumnal bloom of this group was either absent or very small. The difference of the spatial distribution of the *P. delicatissima* and *P. seriata* groups suggests markedly different affinity for different water masses and/or environmental conditions within this one genus. In light of the potential for different levels of toxicity of the different groups (or species within these groups [40] this suggests that the current practice within the Scottish and other harmful phytoplankton monitoring programmes of enumerating *Pseudo-nitzschia* as a single group may be insufficient.

### Patterns of environmental characteristics in relation to phytoplankton distribution

Our cruise was conducted in autumn, a time when productivity remains relatively high in the region, as mixing processes begin to break down summer stratification, making new nutrients available, a process that was most clearly observed at station 2G that was represented by a single point on the T/S diagram (Figure 3). Both physical/chemical conditions and the phytoplankton assemblage were far from homogeneous along the cruise track, with the PCA demonstrating that the ratio of inorganic nutrients DIN∶DSi was the most significant environmental parameter in separating oceanic and shelf stations. The clear diagonal trajectory of sites in the PCA ordination indicates that no one process dominated shelf environmental conditions during the cruise, with the statistically significant correlation of DIN∶DSi and salinity, temperature and density suggesting that physical factors governed nutrient concentrations. Hence, a balance of coastal inputs (both natural and anthropogenic), cross shelf transport, and mixing processes will have acted to influence temperature, salinity and density, associated nutrient concentrations, and as discussed below, phytoplankton community structure.

Comparison of the ordination within the species based MDS and the environment based PCA indicated locational differences in the community composition were generally consistent with those in their environment, and hence that water mass driven environmental conditions govern species distribution, particularly in terms of on/off shelf pattern, but also across the shelf.

Somewhat at odds with the locational pattern of community composition in the MDS is the position of station 9G, mid-way between the shelf and ocean stations, particularly as the PCA did not indicate this station to be exceptionally distinct from the other shelf stations. However, the T/S plot (Figure 3) indicates that, of the shelf stations, the water mass at 9G is most similar to the oceanic stations. Therefore, oceanic water may have transported species found at stations M and F to the 9G area of the shelf. Consistent with this was the observation that the phytoplankton community at 9G did not adhere to the general on/off shelf picture of dinoflagellate distribution, with small dinoflagellates being elevated in concentration at this location in comparison with other shelf stations, demonstrating the potential for physical processes to transport phytoplankton populations as well as nutrients. The greater similarity of station 9G, rather than the further offshore station 10G, to the oceanic water is initially somewhat counter intuitive. However, shelf stations sampled during this study lie in a region extending from Scottish mainland to an area off the southern tip of the Outer Hebrides (Figure 1). Water in this inner region of the shelf is subject to the action of the Scottish coastal current, a northwards flowing stream of reduced salinity water originating from the Irish and Clyde Seas to the south [49]. This coastal current diverges with part continuing northward through the Minch and the remainder flowing in a current initially southwards, along the east coast of the Outer Hebrides, until it rounds Barra Head at the southern tip of the Outer Hebrides and continues northwards. As a result, a gyre connects surface water at station 10G with more coastal water lying approximately between stations 1G and 4G [50], [51] (Figure 1).

Consistent with the hypothesized role of water mass transported nutrients and cells acting together to govern community composition, is the relationship between salinity and DIN∶DSi in the RDA analyses; with salinity significantly related to whole community composition, and both salinity and DIN∶DSi to diatoms alone. Salinity acting as a marker of different water masses is in line with previous studies that have demonstrated high salinity oceanic water to act as a source of DIN for production across the Hebridean shelf break [52]. Similarly, work in the more southerly part of the region has demonstrated a sharp horizontal salinity gradient, associated with the Islay front, to be associated with differences in phytoplankton community composition [53]. Savidge and Lennon [35] also observed a salinity boundary separating coastal and shelf waters, and associated phytoplankton, to the west of Barra Head (to the west of station 10).

The greater abundance of dinoflagellates in high DIN∶DSi off shelf water is consistent with Officer and Ryther [54] who highlighted the potential role of this ratio in governing the balance of diatoms and dinoflagellates. However, as species specific differences exist in diatoms DIN and DSi requirements [41] small changes in nutrient concentration may lead to changes in the DIN∶DSi ratio sufficient to influence the form of nutrient limitation experienced by members of the diatom community itself [55], [56]. Modelling suggests that such changes in relative nutrient availability can influence the composition of a diatom population [57], [58] and is consistent with the relationship between DIN∶DSi and diatom community structure in our RDA. Moreover, when considering the shelf stations alone, RDA analysis demonstrated a close relationship between the salinity and DIN∶DSi ratio with the most abundant single species enumerated in our study, *Lauderia annulata*. This is consistent with this species being most abundant in the surface waters (<30 m) of stations 4G, where the observed DIN∶DSi ratio was also at its lowest (Figure 5a) and with results elsewhere that found this species to dominate at low DIN∶DSi ratios in 11 species competition experiments [59].

Although the conventional view is that a diatom-based food chain is benign [54], changes in the abundance of diatom species may lead to the dominance of harmful genera such as *Pseudo-nitzschia*. The RDA analysis (Figure 11b) suggests that the *P. delicatissima* group was well adapted to the high DIN∶DSi conditions found off the shelf. Consistent with this is the observation that this group is typically found in coastal waters in spring [33] when the DIN∶DSi ratio is high. The relative abundance of different nutrients including DIN and DSi are also thought to be important in determining the toxicity of those *Pseudo-nitzschia* species that produce DA (as it is a nitrogen containing amino acid). Hence DIN stressed/growth limited cells do not produce DA, while stress by other nutrients such as DSi or DIP [60] and iron [61] may increase toxicity. For example, Fehling et al. [62]–[64] demonstrated toxicity of *Pseudo-nitzschia seriata* from Scottish waters, under both DSi and DIP stress (but not DIN stress), observing greater concentrations of the toxin when DSi was in least relative supply. While the nutrient ratio that generates a switch to DSi or DIP rather that DIN limitation of *Pseudo-nitzschia* is not fully established, if we assume that balanced growth would be achieved with a “Redfield” DIN∶DSi∶DIP ratio of 16∶16∶1 [41], [65] then N limitation (and absence of toxin production) would only be expected in the surface waters at stations 4G, 6G and 9G, although station 1G exhibits approximately balanced DIN∶DSi ratios at all depths. One might therefore expect DA to be produced by the toxic members of the *Pseudo-nitzschia seriata* group at other locations and depths. Moreover, the ratio of DSi∶DIP varied between 6.5∶1 and 14.2∶1 on the transect, suggesting that DSi rather than DIP limitation was most likely. This would enhance toxin production [62], and when coupled with the slow depuration of toxin from scallops may underpin the near continual presence of DA in wild harvested scallops from the region.

## Supporting Information

File S1 **Full taxonomic data set.**
(XLS)
